# Understanding Surgery Refusal in Patients with Colorectal Liver Metastases

**DOI:** 10.1007/s12029-026-01503-0

**Published:** 2026-06-22

**Authors:** Laerke Wolf Andreasen, Lucas Alexander Knøfler, Delal Akdag, Sophie Bull Nordkild, Hans-Christian Pommergaard

**Affiliations:** 1 Department of Digestive Diseases, Transplantation and General Surgery, Copenhagen University Hospital, Rigshospitalet, Copenhagen, Denmark; 2 Hepatic Malignancy Surgical Research Unit (HEPSURU), Department of Digestive Diseases, Transplantation and General Surgery, Copenhagen University Hospital, Rigshospitalet, Copenhagen, Denmark; 3 Institute for Clinical Medicine, University of Copenhagen, Panum Institute, Copenhagen, Denmark; 4 Section for Enhanced Recovery after Surgery and Disease (ERASD), Copenhagen University Hospital, Rigshospitalet, Copenhagen, Denmark

**Keywords:** Refusal, CRLM, Liver metastases, Reasons, Risk factors, Survival

## Abstract

**Purpose:**

To investigate the risk factors, reasons, and survival outcomes associated with refusal of surgery for colorectal liver metastases (CRLM). Surgery is the only curative treatment for CRLM, however, some patients refuse. The risk factors and reasons for refusal remain uninvestigated in patients with CRLM.

**Methods:**

Patients with resectable and operable CRLM referred for treatment at Copenhagen University Hospital (2013–2023), were identified through the Danish Liver Cancer Group registry. Those who refused surgery were matched 1:4 by WHO performance status with patients who accepted surgery. Data were retrieved from medical records. Risk factors were evaluated using Conditional Logistic Regression. Reasons for refusal were described by frequencies. Survival was analyzed using the Kaplan-Meier estimator and Cox Regression.

**Results:**

Among 2,727 patients referred for treatment, 1,478 were resectable and operable. Twenty-one patients (1.4%) refused surgery. Lower BMI and longer time since the primary tumor diagnosis increased the odds of refusing surgery by 15% per kg/m^2^ (95% CI 0.76–0.99) and 3% per month (95% CI 1.01–1.06), respectively. Reasons for refusal included declining further surgery, logistical priorities, preference for chemotherapy and self-assessed unfitness. Patients who refused surgery had significantly lower overall survival (*p* < 0.003) and higher mortality risk (HR: 1.87; 95% CI: 1.02–3.43).

**Conclusion:**

This study is the first to identify risk factors and reasons for refusal of surgery in CRLM. The findings provide insight into motivational and prognostic considerations that may support shared decision-making. Further research across cancer types is needed to optimize patient care.

## Introduction

### Background

Colorectal cancer is the third most common cancer and the second leading cause of cancer-related deaths globally [1]. The most common site of metastases is the liver [2]. During their illness, 25–50% of patients with colorectal cancer develop liver metastases, which are associated with reduced long-term survival [3, 4]. Hepatic resection remains the gold standard and, in combination with adjuvant chemotherapy, the 5-year survival rate exceeds 50% [3]. In the absence of surgery, due to either non-resectability or non-operability, the 5-year survival rate approaches zero [3, 5].

Some patients refuse surgery despite being deemed resectable and operable. The risk factors contributing to surgery refusal remain uninvestigated among patients with colorectal liver metastases (CRLM). In other cancer types, frequently cited risk factors of treatment refusal include age, race, sex, disease stage, comorbidity, healthcare insurance, and the level of the cancer treatment facility [6, 7]. However, the individual personal reasons for refusing surgery remain largely unknown. A study from 2005 on patients with breast cancer identified the following reasons: psychiatric issues, pursuit of alternative therapy, other medical conditions, fear of surgery, and perceiving themselves as too old [8]. Understanding the characteristics of patients who refuse surgery, and their reasoning could improve patient care.

The primary objective of this study was to identify risk factors and reasons for refusing surgical treatment of CRLM in a Danish high-volume Hepato-Pancreato-Biliary center. The secondary objective was to investigate the overall survival and the hazard of mortality following the refusal of surgical treatment compared to patients undergoing surgery for CRLM.

## Methods

### Study Design and Settings

We conducted a single-center, matched case-control study using data from the Danish Liver Cancer Group (DLGCD) registry, maintained by The Danish Clinical Quality Program – National Clinical Registries (RKKP). The DLGCD registry contains patient information from Multidisciplinary Team (MDT) conferences, where care plans are formulated collaboratively by surgeons, oncologists, hepatologists, radiologists and interventional radiologists. Patients planned for surgery undergo an operability assessment by a surgeon and the surgical treatment plan is subsequently presented to the patients by a senior surgeon. Patients are informed about the risks and benefits of surgery, including the risk of recurrence, overall prognosis, and details of the surgical procedure. This study included patients referred for MDT conferences between March 2013 and December 2023 at Copenhagen University Hospital, Rigshospitalet. It is the largest of four Hepato-Pancreato-Biliary centers in Denmark, covering approximately half the population. Patients who refused surgery were matched to controls who accepted surgery. Registry data were supplemented with information from medical records and stored using Research Electronic Data Capture (REDCap).

Ethical approval for data use and storage was obtained from The Capital Region of Denmark (R-23057752, P-2024-15920).

### Study Population

We included all patients with CRLM, who were initially deemed operable and resectable by a referring physician and subsequently offered surgery after an MDT conference, but without a date of surgery in the registry, indicating that no surgery was performed. Surgery included single- and two-stage liver resections, ablation, or a combination of liver resection and ablation. We excluded patients who did not undergo surgery for reasons other than patient refusal, including subsequent physician evaluation at Rigshospitalet deeming the patient inoperable due to comorbidity, health decline, or disease progression. Patient refusal was defined as the patient independently choosing not to undergo surgery, despite physician recommendation.

We matched patients in a 1:4 ratio, based on WHO performance status (PS) at the time of referral to the MDT conference to ensure comparability in frailty [9]. Controls were patients with CRLM who were initially deemed operable and resectable by the referring physician, with an MDT plan of surgery, and a subsequent registered surgery date in the DLGCD registry.

### Definitions

Data from medical records were retrieved from the pre-surgical consultations following the MDT conference, where surgery was subsequently refused (cases) or the last MDT where surgery was accepted (controls).

Baseline characteristics included: age (at MDT), sex, Body Mass Index (BMI), PS at preoperative assessment (which was not equal to the PS used for matching), civil status, American Society of Anesthesiologists grade of physical status (ASA), Charlson Comorbidity Index (CCI), psychiatric comorbidity, stoma, previous abdominal surgery (including all abdominal surgeries), prior MDT conferences (including all cancer types), time from facility (travel time by car from home address to Rigshospitalet), months from primary tumor and hepatic metastases (from date of diagnosis in pathology and computer tomography imagining (CT), respectively), and Carcinoembryonic Antigen (CEA).

Tumor characteristics, recorded from pathology reports and CT descriptions, included: tumor sequence, number of hepatic tumors, size of largest hepatic tumor, extrahepatic, mutations, and location of primary tumor.

Oncological summaries provided information regarding resection of primary tumor, adjuvant therapy after primary tumor, neoadjuvant therapy for CRLM, and previous radiation treatment.

The type of surgery offered at MDT was registered from conference notes and surgical plans. Reasons for refusals were recorded in full details, and categorized as: declining further surgery, logistical priorities, preference for chemotherapy, no further treatment, self-assessed unfitness, or other.

Survival time was defined as time from the last preoperative MDT until death from any cause or last follow-up (6th of November 2024).

### Statistical Analysis

Descriptive statistics were reported as frequencies and percentages for categorical variables. Continuous variables were reported as means and standard deviations (SD) for normally distributed data, and as medians with interquartile ranges (IQR) for non-normally distributed data. The normality of distributions was assessed using the Shapiro-Wilk test.

Matching of cases and controls was performed using nearest-neighbor matching without replacements, in a 1:4 ratio of cases to controls.

A conditional logistic regression model was developed to estimate the odds of refusing surgery associated with prespecified preoperative factors. Model estimates were described as odds ratios (OR) with corresponding 95% confidence intervals (CI). All variables were assessed exploratorily in univariable analyses and included for multivariable analysis if the estimates were deemed statistically probable at a p-value < 0.05. A multivariable conditional logistic regression model, adjusted for BMI, CCI, and months from primary tumor diagnosis, was proposed. The goodness of fit of the model was assessed regarding linearity on log-odds scale, multicollinearity, and the impact of outliers in data. Outliers were found in BMI, CCI, and months from primary tumor diagnosis. These outliers were assessed for correctness and deemed clinically representative and of low impact for model estimates and were therefore kept for the analysis. No multicollinearity or violation of the linearity assumption was detected.

A Cox proportional hazards regression model was conducted to assess the effect of surgery refusal compared to acceptance on mortality risk. Model estimates were described as hazard ratios (HR), with corresponding 95% CI’s. All variables were included in the univariable analysis. Due to the limited sample size, only variables with a significance level of *p* < 0.01 were included in the multivariable analysis. The variables included in the multivariable Cox regression were refusal of surgery, CCI, and ASA. There was no interaction between CCI and ASA. The goodness of fit of the model was assessed for linearity and proportionality using Martingale residuals. ASA did not meet the proportionality assumption; therefore, a stratification approach was applied to address this. The concordance index stood at 0.59 (SE = 0.044).

Long-term survival was described using the Kaplan-Meier estimator. The estimator was stratified by cases and controls and compared using the log-rank test.

Statistical significance was considered as a p-value < 0.05. All statistical analysis was performed using R studio and R version 4.4.1.

## Results

### Patient Characteristics

**Fig. 1:**
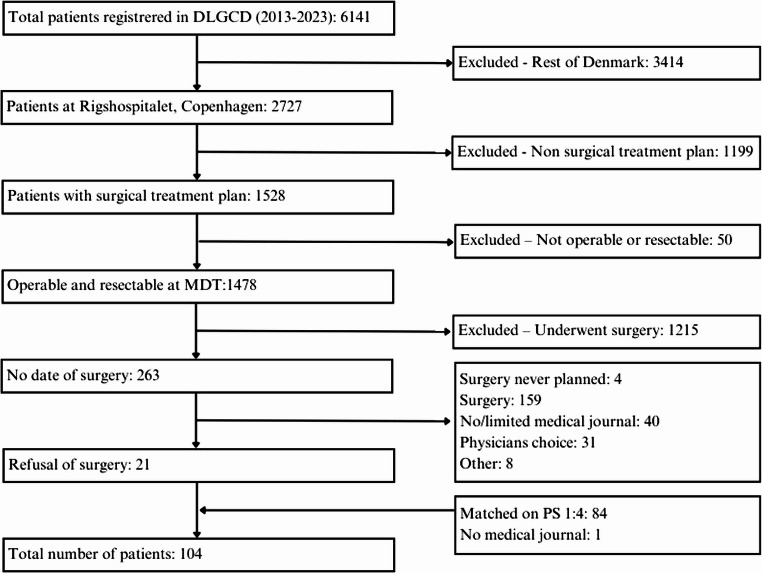
Identification of patients who refused surgery. Cases were matched in a 1:4 ratio with patients who accepted surgery

A total of 6,141 patients were registered in the DLGCD registry. Among them, 2,727 patients (44%) were treated at Copenhagen University Hospital, Rigshospitalet. Of these, 263 patients met the inclusion criteria. Upon reviewing the medical records, most patients were excluded (Fig. 1). The 21 cases were matched with 83 controls who accepted surgery (one patient was excluded due to missing medical records).

The rate of patient refusal at Copenhagen University Hospital, Rigshospitalet was 1.4%.

The 104 patients included in the study had a median age of 67 years. Most were male (63%), with a median BMI of 26. The majority (69%) were married or living with a partner. The median CCI was 9, as all patients had metastatic disease. The median time from primary tumor diagnosis was 13 months, with a median of 5 months since the first liver metastases. The most frequent primary tumor location was the rectum (35%) or the sigmoid colon (24%). One-third of the patients had simultaneous extrahepatic disease, mainly due to primary tumor in situ. Most patients had a history of previous abdominal surgery, including resection of the primary tumor. Seventy-one patients (68%) had no history of prior liver surgery. Clinical characteristics at baseline are summarized in Table 1.

**Table 1:** Patient characteristics

	Refuses *N* = 21	Accepts *N* = 83	Overall *N* = 104
Age, mean (SD)	69 (± 9)	66 (± 10)	67 (± 10)
Sex, n (%)			
Female	6 (29)	33 (40)	39 (38)
Male	15 (71)	50 (60)	65 (63)
BMI, median (IQR) Missing	25 (23–27) 1	27 (24–30) 0	26 (23–30) 1
Charlson Comorbidity Index			
Median (IQR)	9 (8–10)	8 (8–10)	9 (8 − 10)
Psychiatric comorbidity, n (%)			
No	17 (81)	75 (90)	92 (88)
Yes	4 (19)	8 (9.6)	12 (12)
ASA, n (%)			
1	3 (15)	23 (28)	26 (25)
2	11 (55)	45 (54)	56 (54)
3	6 (30)	15 (18)	21 (20)
Missing	1	0	1
Performance Status preoperative assessment, n (%)			
0	11 (52)	58 (70)	69 (66)
1	7 (33)	21 (25)	28 (27)
2	3 (14)	4 (4.8)	7 (6.7)
> 2	0	0	0
Civil Status, n (%)			
Married	13 (62)	59 (71)	72 (69)
Single	6 (29)	23 (28)	29 (28)
Widowed within 5 years	2 (9.5)	1 (1.2)	3 (2.9)
Time from facility, n (%)			
< 1 h	18 (86)	64 (77)	82 (79)
> 1 h	3 (14)	19 (23)	22 (21)
Months from primary tumor, median (IQR)	20 (8–43)	12 (5–25)	13 (6–25)
Location of primary tumor, n (%)			
Rectum	9 (38)	30 (34)	39 (35)
Sigmoid colon	7 (29)	20 (23)	27 (24)
Descending colon	1 (4)	7 (8)	8 (7)
Transverse colon	4 (17)	9 (10)	13 (12)
Ascending colon	1 (4)	6 (7)	7 (6)
Coecum	2 (8)	12 (14)	14 (13)
Other	0	3 (3)	3 (3)
Unknown	0	1	1
Mutations in primary tumor, n (%)			
No	10 (48)	47 (57)	57 (55)
Yes	11 (52)	36 (43)	47 (45)
KRAS	9 (43)	26 (31)	35 (34)
BRAF	1 (5)	11 (13)	12 (12)
NRAS	1 (5)	4 (5)	5 (5)
PIK3CA	1 (5)	2 (2)	3 (3)
pMx	1 (5)	0	1 (1)
Previous abdominal surgery,			
Median (IQR)	2 (1–3)	2 (1–3)	2 (1–3)
Resection of primary tumor, n (%)			
No	5 (24)	24 (29)	29 (28)
Yes	16 (76)	59 (71)	75 (72)
Stoma, n (%)			
No	14 (67)	68 (82)	82 (79)
Yes	7 (33)	15 (18)	22 (21)
Adjuvant therapy after primary tumor, n (%)			
No	13 (62)	53 (64)	66 (63)
Yes	8 (38)	30 (36)	38 (37)
Months from hepatic metastases, median (IQR)	8 (4 − 19)	5 (1–11)	5 (1–13)
Tumor sequence, n (%)			
Metachronous	10 (48)	28 (34)	38 (37)
Synchronous	11 (52)	55 (66)	66 (63)
Previous liver surgery, n (%)			
Median (IQR)	0 (0–1)	0 (0–1)	0 (0–1)
Prior MDT conferences, n (%)			
≤ 4	15 (71)	62 (75)	77 (74)
> 4	6 (29)	21 (25)	27 (26)
Number of hepatic tumors, n (%)			
1	9 (43)	38 (46)	47 (45)
2	7 (33)	12 (14)	19 (18)
3	0	8 (9.6)	8 (7.7)
4	0	5 (6.0)	5 (4.8)
More than 4	5 (24)	20 (24)	25 (24)
Size of largest hepatic tumor (cm), median (IQR) Unknown	2.0 (1.5–3.0) 0	2.3 (1.6–3.1) 1	2.2 (1.5–3.1) 1
Extrahepatic disease, n (%)			
No	14 (67)	57 (69)	71 (68)
Yes	7 (33)	26 (31)	33 (32)
Primary tumor in situ	4 (19)	20 (24)	24 (23)
Lungs	3 (14)	4 (5)	7 (7)
Lymphnodes	1 (5)	2 (2)	3 (3)
Peritoneum	0	2 (2)	2 (2)
Other	2 (10)	4 (5)	6 (6)
CEA (µg/L) – median (IQR) Unknown	7 (4–20) 2	5 (3–11) 16	6 (3–11) 18
Neoadjuvant therapy for CRLM, n (%)			
No	8 (38)	30 (36)	38 (37)
Yes	13 (62)	53 (64)	66 (63)
Previous radiation treatment, n (%) No Yes	18 (86) 3 (14)	74 (89) 9 (11)	92 (88) 12 (12)
Offered surgery: Approach, n (%)			
Laparoscopic	3 (14)	10 (12)	13 (13)
Open	16 (76)	64 (77)	80 (77)
Percutaneous	2 (9.5)	8 (9.6)	10 (9.6)
Robotic	0	1 (1.2)	1 (1.0)
Offered surgery: Method – n (%)			
Resection	13 (62)	53 (64)	66 (63)
Ablation	3 (14)	10 (12)	13 (13)
Combination	3 (14)	11 (13)	14 (13)
Two-stage	0	6 (7.2)	6 (5.8)
Other	2 (9.5)	3 (3.6)	5 (4.8)

Baseline characteristics of patients included in the study. Data is presented in three categories, Refuses Surgery, Accepts Surgery and Overall. All data was gathered around MDT where surgery was offered and either accepted or refused.*IQR* Interquartile range, *n* number, *%* percentages. Missing data is listed.*ASA* American Society of Anesthesiologists grade of physical status. *BMI* Body Mass Index. *CEA* Carcinoembryonic antigen. *CRLM* Colorectal liver metastases. *MDT* Multidisciplinary Team. *pMx* pMLH1, pMSH2, pMSH6 and pPMS2

### Refusal of Surgery

In univariable conditional logistic regression analyses, lower BMI, higher CCI, and more months from primary tumor diagnosis were significantly associated with increased odds of surgery refusal. Specifically, lower BMI was associated with a 13% increase in odds of refusing surgery (OR: 0.87, 95% CI: 0.76–0.99), higher CCI was associated with a 38% increase in odds (OR: 1.38, 95% CI: 1.00–1.91), and every month since primary tumor diagnosis was associated with a 3% increase in odds (OR: 1.03, 95% CI: 1.00–1.05). Univariable estimates are detailed in Table 2.

**Table 2:** Risk factors for refusal of surgery

	Univariable analysis		Multivariable analysis	
	OR (95% CI)	*p*	OR (95% CI)	*p*
Age, per year	1.04 (0.99–1.09)	0.16		
Sex				
Female (ref.)	–			
Male	1.63 (0.58–4.56)	0.35		
BMI, per kg/m^2^	0.87 (0.76–0.99)	0.03*	0.85 (0.74–0.98)	0.03*
Charlson Comorbidity Index, per point	1.38 (1.00–1.91)	0.05*	1.35 (0.94–1.94)	0.11
Psychiatric comorbidity				
No (ref.)	-	-		
Yes	2.23 (0.59–8.39)	0.23		
ASA				
1 (ref.)	-	-		
2	1.88 (0.48–7.32)	0.37		
3	3.32 (0.67–16.41)	0.14		
Civil Status				
Married (ref.)	-	-		
Single	1.21 (0.41–3.54)	0.73		
Widowed	8.74 (0.75–101.64)	0.08		
Time from facility				
< 1 h (ref.)	-			
> 1 h	0.56 (0.15–2.12)	0.39		
Months from primary tumor diagnosis, per month	1.03 (1.00–1.05)	0.03*	1.03 (1.01–1.06)	0.01*
Mutations in primary tumor				
No (ref.)	-			
Yes	1.43 (0.55–3.70)	0.46		
Previous abdominal surgery, per surgery	1.03 (0.78–1.34)	0.85		
Resection of primary tumor				
No (ref.)	-			
Yes	1.31 (0.42–4.07)	0.64		
Stoma				
No (ref.)	-			
Yes	2.22 (0.78–6.37)	0.14		
Adjuvant therapy after primary tumor				
No (ref.)	-			
Yes	1.09 (0.41–2.88)	0.87		
Months from hepatic tumor, per month	1.03 (1.00–1.07)	0.06		
Tumor sequence				
Metachronous (ref.)	-			
Synchronous	0.56 (0.21–1.48)	0.24		
Previous liver surgery, per surgery	1.12 (0.66–1.88)	0.68		
Prior MDT conferences				
≤ 4 (ref.)	-			
> 4	1.18 (0.41–3.39)	0.76		
Number of hepatic tumors				
1 (ref)	-			
2	2.41 (0.75–7.71)	0.14		
3	0–inf.	0.99		
4	0–inf.	0.99		
More than 4	1.03 (0.31–3.49)	0.96		
Size of largest hepatic tumor, per cm	0.90 (0.64–1.26)	0.53		
Extrahepatic disease				
No (ref.)	-			
Yes	1.10 (0.39–3.11)	0.85		
CEA, per µg/L	1.01 (0.99–1.04)	0.23		
Neoadjuvant therapy for CRLM				
No (ref.)	-			
Yes	0.92 (0.34–2.47)	0.87		
Previous radiation treatment				
No (ref.)	-			
Yes	1.36 (0.34–5.37)	0.67		
Offered surgery: Approach				
Laparoscopic (ref.)	-			
Open	0.83 (0.20–3.37)	0.79		
Percutaneous	0.83 (0.12–6.01)	0.86		
Robotic	0–inf.	0.99		
Offered surgery: Method				
Ablation (ref)	-			
Combination	0.91 (0.15–5.48)	0.92		
Other	2.15 (0.24–19.09)	0.49		
Resection	0.82 (0.20–3.32)	0.78		
Two-stage	0–inf.	0.99		

Risk factors for refusal of surgery were investigated using univariable and multivariable conditional logistic regression. Only variables with *p* ≤ 0.05* were included for the multivariable analysis. All data was gathered around MDT where surgery was offered and either accepted or refused.*ASA* American Society of Anesthesiologists grade of physical status. *BMI* Body Mass Index. *CEA* Carcinoembryonic antigen. *CRLM* Colorectal liver metastases. *MDT* Multidisciplinary Team

In multivariable conditional logistic regression analysis, lower BMI and more months from primary tumor diagnosis remained significant risk factors for refusal of surgery. Specifically, lower BMI was associated with a 15% increase in odds of refusing surgery (OR: 0.85, 95% CI: 0.74–0.98), and every month since primary tumor diagnosis was associated with 3% increased odds (OR: 1.03, 95% CI: 1.01–1.06). CCI did not retain statistical significance after adjustment. Moreover, there was no statistical interaction between CCI and BMI.

### Survival Analysis

**Fig. 2:**
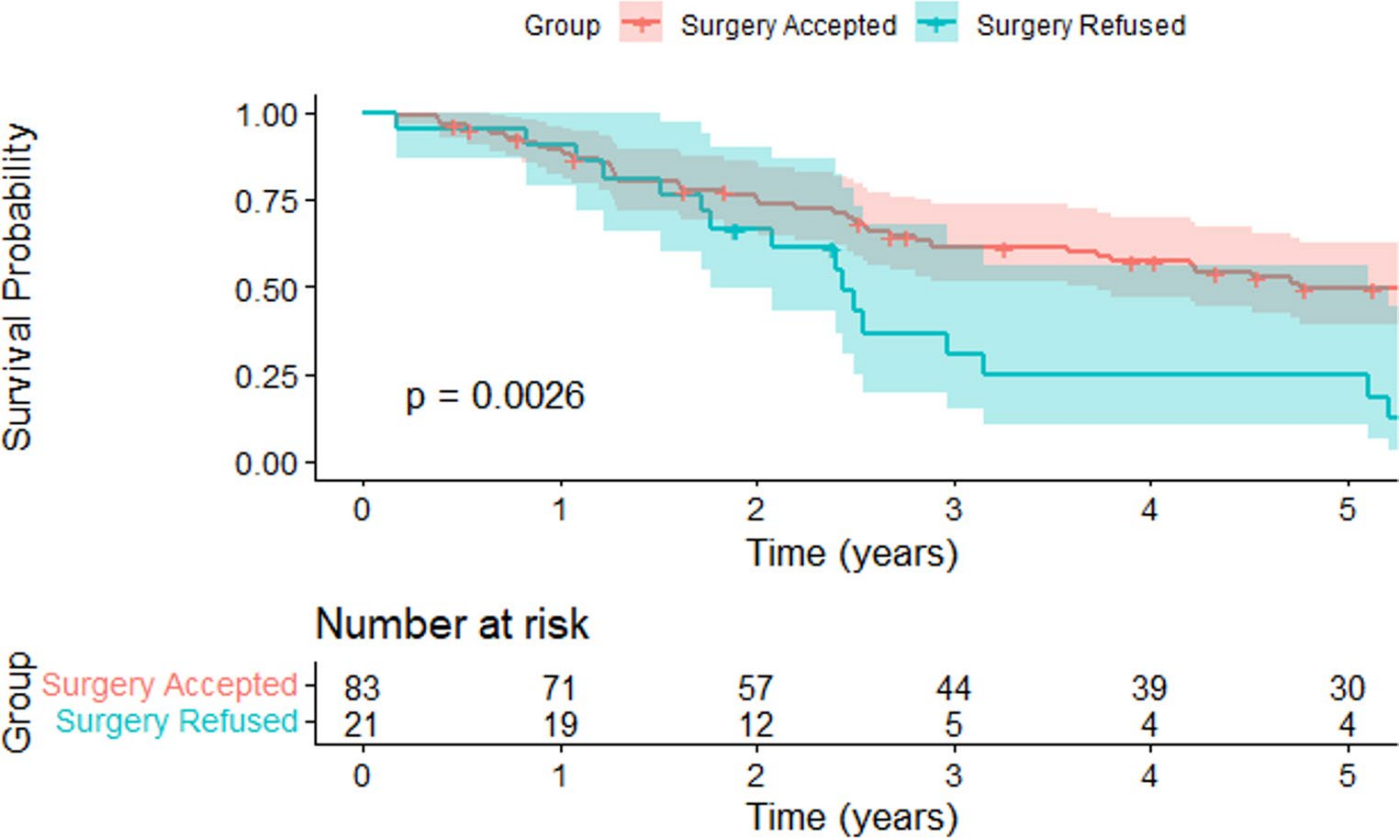
Overall survival calculated using Kaplan-Meier. *Time(years): measured from MDT conference where surgery was refused (cases) or last MDT conference where surgery was accepted (controls)*

Patients who refused surgery had significantly lower long-term survival compared to those who accepted surgery. The 5-year survival probabilities were 19% for patients who refused surgery and 36% for those who accepted surgery. The median survival time for patients who refused surgery was 2.44 years (95% CI: 1.77–5.21), whereas the patients who accepted surgery had a median survival time of 4.76 years (95% CI: 3.73–9.02) (Fig. 2).

In univariable Cox regression analysis, refusing surgery was associated with an increased mortality risk (HR: 2.37, 95% CI: 1.33–4.24). Other significant risk factors included lower BMI (HR: 0.94, 95% CI: 0.89–0.99), ASA score of 3 (HR: 2.80, 95% CI: 1.32–5.96), higher CCI (HR: 1.33, 95% CI: 1.13–1.57), months since primary tumor diagnosis (HR: 1.01, 95% CI: 1.00-1.02), having a stoma (HR: 1.84, 95% CI: 1.03–3.29), elevated CEA levels (HR: 1.01, 95% CI: 1.00-1.01), and receiving neoadjuvant chemotherapy (HR: 1.81, 95% CI: 1.04–3.13).

Multivariable Cox regression analysis, limited to risk factors with *p* < 0.01 due to the small sample size, identified higher CCI (HR: 1.25, 95% CI: 1.04–1.51) and refusing surgery (HR: 1.87, 95% CI: 1.02–3.43) as risk factors of increased mortality risk. The model was stratified by ASA Table 3.

**Table 3:** Hazard of mortality

	Univariable analysis		Multivariable analysis	
	HR (95% CI)	*p*	HR (95% CI)	*p*
Age, per year	1.02 (1.00–1.05)	0.10		
Sex				
Female (ref.)	-			
Male	1.08 (0.65–1.80)	0.77		
BMI, per kg/m^2^	0.94 (0.89–1.00)	0.04*		
Charlson Comorbidity Index, per point	1.33 (1.13–1.57)	<0.01**	1.25 (1.04–1.51)	0.02*
Psychiatric comorbidity				
No (ref.)	-			
Yes	1.51 (0.74–3.06)	0.26		
ASA				
1 (ref.)	-			
2	1.50 (0.79–2.86)	0.22		
3	2.80 (1.32–5.96)	<0.01**		
Civil Status				
Married (ref.)	-			
Single	1.07 (0.61–1.85)	0.82		
Widowed	2.29 (0.71–7.44)	0.17		
Time from facility				
< 1 h (ref.)	-			
> 1 h	0.77 (0.41–1.44)	0.41		
Months from primary tumor diagnosis, per month	1.01 (1.00–1.02)	0.05*		
Mutations in primary tumor				
No (ref.)	-			
Yes	1.17 (0.71–1.92)	0.54		
Previous abdominal surgery, per surgery	1.06 (0.93–1.22)	0.38		
Resection of primary tumor				
No (ref.)	-			
Yes	1.16 (0.67–2.00)	0.60		
Stoma				
No (ref.)	-			
Yes	1.84 (1.03–3.29)	0.04*		
Adjuvant therapy after primary tumor				
No (ref.)	-			
Yes	1.04 (0.62–1.74)	0.88		
Months from hepatic tumor, per month	1.01 (1.00–1.03)	0.12		
Tumor sequence				
Metachronous (ref.)	-			
Synchronous	0.65 (0.39–1.08)	0.10		
Previous liver surgery, per surgery	1.16 (0.88–1.51)	0.29		
Prior MDT conferences				
≤ 4 (ref.)	-			
> 4	1.37 (0.73–2.40)	0.27		
Number of hepatic tumors				
1 (ref)	-			
2	1.46 (0.76–2.83)	0.26		
3	0.81 (0.28–2.33)	0.69		
4	1.46 (0.51–4.22)	0.48		
More than 4	1.43 (0.76–2.70)	0.27		
Size of largest hepatic tumor, per cm	1.10 (0.93–1.30)	0.26		
Extrahepatic disease				
No (ref.)	-			
Yes	1.10 (0.66–1.83)	0.73		
CEA, per µg/L	1.01 (1.00–1.01)	0.04*		
Neoadjuvant therapy for CRLM				
No (ref.)	-			
Yes	1.81 (1.04–3.13)	0.04*		
Previous radiation treatment				
No (ref.)	-			
Yes	0.69 (0.30–1.59)	0.38		
Offered surgery: Approach				
Laparoscopic (ref.)				
Open	-			
Percutaneous	1.90 (0.81–4.43)	0.14		
Robotic (NA)	1.61 (0.49–5.31)	0.43		
Offered surgery: Method				
Ablation (ref)	-			
Combination	1.18 (0.46–3.05)	0.73		
Other	0.56 (0.14–2.16)	0.40		
Resection	0.70 (0.31–1.58)	0.40		
Two-stage	2.21 (0.69–7.06)	0.18		
Surgery Accepted or Refused				
Accepted (ref.)	-		-	
Refused	2.37 (1.33–4.24)	<0.01**	1.87 (1.02–3.43)	0.04*

Risk of mortality calculated using cox proportional hazard regression. Due to the number of events only variables with a significance level of *p* < 0.01** were included in the multivariable analysis. *CCI* ASA and Refusal of surgery were included for the multivariable analysis. The model was stratified by ASA.*ASA* American Society of Anesthesiologists grade of physical status. *BMI* Body Mass Index. *CEA* Carcinoembryonic antigen. *CRLM* Colorectal liver metastases. *MDT* Multidisciplinary Team

### Reasons for Refusal

The most common reason for refusing surgery was declining further surgery (*n* = 8). Some patients had previously experienced complicated surgeries and cited this as a reason to avoid additional surgical treatment. Other patients specifically declined open surgery or accepted only radiofrequency ablation.

Another reason was logistical priorities (*n* = 3), such as a long-term family vacation or being unable to undergo surgery at that time. Preference for chemotherapy (*n* = 3) included one patient who hoped it would reduce the extent of surgery. Some patients wanted no further treatment for their disease (*n* = 2), while others refused due to self-assessed unfitness (*n* = 2).

Other reasons included anxiety (*n* = 1), treatment abroad (*n* = 1), and alternative therapy (*n* = 1).

### Treatment After Refusal

The majority of patients were treated with chemotherapy after refusal (*n* = 9), or no treatment at all (*n* = 8). The remaining patients chose either stereotactic radiotherapy (*n* = 1), alternative therapy (*n* = 1), treatment abroad (*n* = 1), or radiofrequency ablation (*n* = 1). Four patients, later in their disease, opted for surgery for their liver metastases.

## Discussion

To our knowledge, this study is the first to investigate the risk factors and reasons for refusing surgery in patients with liver metastases, providing insights beyond those typically derived from registry studies.

### Risk Factors for Refusal of Surgery

Patients who refused surgery were more likely to have a lower BMI and a longer duration since their primary tumor diagnosis. Lower BMI has been associated with poorer overall survival in CRLM, with the proposed mechanism being cancer cachexia [10]. However, this mechanism is not fully understood, and low BMI may instead reflect frailty, sarcopenia, or cachexia. Studies from the National Cancer Database and the Surveillance, Epidemiology, and End Results Database across cancer types have shown that refusal was more common among older, unmarried, non-white ethnic patients, those with higher comorbidity burden, or insufficient insurance coverage [6-8, 11-21]. Denmark’s universal healthcare system eliminates insurance status as a risk factor for surgery refusal, which is often significant in other healthcare settings [6, 7, 11-14, 16, 19, 20]. The refusal rate in this study is equivalent to colorectal cancer surgery refusal rates, reported at 0.6–3.5% [6, 17].

Unlike most registry-based studies, our research utilized detailed patient records, enabling a more nuanced understanding of risk factors. However, this reliance on records limited our sample size and statistical power.

### Reasons for Refusal of Surgery

Improved understanding of surgical refusal may facilitate shared decision-making, as awareness of patient concerns enables physicians to address them more effectively. In our study, a substantial proportion of refusals appeared related to treatment fatigue, with the primary reason being declining further surgery. This is further supported by the number of patients choosing no further treatment or opting for palliative chemotherapy. The reasons for refusal were comparable to a breast cancer study, finding that alternative therapy, while easy to blame for patients refusing standard healthcare, was not the only nor main reason [8]. However, CRLM patients were more likely to cite cumulative treatment burden and treatment fatigue compared to breast cancer patients, where psychiatric comorbidity was the main factor. This difference likely reflects the advanced disease stage and extensive treatment history of patients with CRLM.

Interestingly, disease intensity, as indicated by the number of MDT conferences, prior treatments, and surgeries, was not associated with increased refusal rates. Instead, longer disease duration influenced refusal, indicated by more months since primary tumor diagnosis. However, prior experiences with complicated surgeries, with several patients citing aversion to large or open surgery and hoping for a smaller intervention. These findings suggest that advancements in minimally invasive surgical techniques could address the needs of patients currently refusing surgery.

### Overall Survival and the Hazard of Mortality Following the Refusal of Surgery

Accepting surgery resulted in a median survival time of 4.76 years, comparable to that observed in a similar Norwegian cohort (median survival 4.78 years) [22]. In our analysis, refusal of surgery was associated with poorer survival outcomes among patients with CRLM, corresponding to a median survival difference of 2.3 years. The survival curves diverged after approximately two years, which may be clinically relevant when counselling patients about the potential benefits of surgical treatment. However, due to the limited number of events, subgroup analyses assessing the differential impact of post-refusal treatment strategies, including delayed surgery, were not feasible.

Multivariable cox regression analysis showed that refusal of surgery almost doubled the risk of mortality. This finding remained significant when adjusting for CCI and ASA. While residual confounding, such as increased frailty not captured by CCI or ASA, cannot be excluded, comparable tumor characteristics between groups suggest that refusal itself may impact survival. These findings may contribute to more informed discussions with patients regarding expected prognosis and the potential consequences of refusing surgery.

This impact of refusing surgery was also found across cancer types. In CRC, refusing surgery was associated with an increase in mortality risk (HR: 2.32–5.98) [11, 12, 14]. In other cancer types, including hepatocellular carcinoma, breast cancer, head and neck and ovarian cancer, the same pattern was seen (HR: 2.42–2.88) [15, 16, 18, 20].

### Strengths, Limitations and Impact

The retrospective design of this study enabled detailed data collection. However, the principal limitation is the small sample size, which reduces statistical power across all analyses. It also limited our ability to include additional prognostic variables in the Cox regression model—such as tumor size, CEA level, age, presence of extrahepatic metastases, and KRAS status—as including multiple variables would have resulted in model overfitting. The small sample size, due to the rarity of surgery refusal and the number of cases available over the 10-year study period, may also have restricted the detection of some risk factors. Although not statistically significant, baseline characteristics suggest that the likelihood of refusal increases with factors such as older age, comorbidity burden, psychiatric disorders, and the loss of a partner.

There was a discrepancy between the referral performance status (PS) recorded in the dataset used for patient matching and the preoperative PS documented in the patient records. Baseline characteristics showed that a higher proportion of patients in the refusal group had poorer PS compared with those who accepted surgery, possibly reflecting selection bias. However, PS at the preoperative assessment was not available in the database used for matching controls. Although data extraction from medical records enabled a high level of detail and improved data validity, missing or inaccurately recorded information remains a potential limitation.

Furthermore, this study was done retrospectively, reducing the quality of the qualitative outcomes, and making it impossible to further investigate the reasons other than what is recorded in medical journals, as most patients who refused surgery were dead. However, this study cannot be done prospectively, as the occurrence of refusal is so rare. There is a risk of residual confounding, as the cohort is small, the poorer survival might not be due to refusal, but rather the differences between the groups that we cannot adjust for.

As the incidence is small, individual physicians will not encounter many patients refusing treatment, so investigating these factors will help better physicians’ knowledge. Quantifying the impact of refusal on survival has not been done before in CRLM. Understanding reasons for surgery refusal and its impact on survival will help the physician provide individual guidance in the preoperative decision-making. It also challenges the misconception that patients who refuse conventional medicine, are prone to select alternative therapy.

While individual numbers for each cancer type are small, the aggregate number of patients refusing treatment across cancer types highlights the importance of understanding these factors. Further exploration includes better and more in-depth registration of reasons behind refusal in databases, not only in liver surgery but across cancer diseases. This will enable us to adjust treatment according to patient preferences, for example avoiding open surgery if possible or increasing neoadjuvant therapy to reduce the surgical burden. Investigations should strive to make comparisons across cancer types, to gain an understanding of which factors are transferable and make a larger impact in this rare area.

## Conclusion

This study was the first to investigate risk factors for refusal of surgery in patients with CRLM. It is the second study, to our knowledge to investigate the reasons behind refusal for any cancer type. Risk factors for refusal of surgery were lower BMI and longer time since diagnosis of the primary tumor. The main reasons for refusal included declining further surgery, self-assessed unfitness, treatment fatigue, and a preference for chemotherapy. Refusing surgery significantly reduced survival, providing new insight into its impact on prognosis.

These findings may help physicians better address patient preferences and provide information regarding the expected impact on survival, ultimately optimizing shared decision-making. Further investigation into risk factors and reasons for refusal is needed across cancer types. Such studies should go beyond database-driven designs to capture nuanced patient perspectives.

## Data Availability

Data from patient records and the Danish Liver Cancer Group were used in this study. Due to restrictions on patient data, these datasets are not publicly available but may be available from the authors upon reasonable request.
